# Standardizing and Scaffolding Health Care AI-Chatbot Evaluation: Systematic Review

**DOI:** 10.2196/69006

**Published:** 2025-11-07

**Authors:** Yining Hua, Winna Xia, David Bates, George Luke Hartstein, Hyungjin Tom Kim, Michael Li, Benjamin W Nelson, Charles Stromeyer IV, Darlene King, Jina Suh, Li Zhou, John Torous

**Affiliations:** 1 Technology and Operations Management Harvard Business School Cambridge, MA United States; 2 Department of Psychiatry Beth Israel Deaconess Medical Center Boston, MA United States; 3 Division of Internal Medicine Brigham and Women's Hospital Boston, MA United States; 4 Department of Psychiatry Thomas Jefferson University Philadelphia, PA United States; 5 Department of Psychiatry and Human Behavior Brown University Providence, RI United States; 6 Department of Psychiatry University of Texas Southwestern Houston, TX United States; 7 Microsoft (United States) Redmond United States

**Keywords:** large language model, artificial intelligence, AI chatbots, evaluation framework, generative AI

## Abstract

**Background:**

Health care chatbots are rapidly proliferating, while generative artificial intelligence (AI) outpaces existing evaluation standards.

**Objective:**

We aimed to develop a structured, stakeholder-informed framework to standardize evaluation of health care chatbots.

**Methods:**

PRISMA (Preferred Reporting Items for Systematic reviews and Meta-Analyses)–guided searches across multiple databases identified 266 records; 152 were screened, 21 full texts were assessed, and 11 frameworks were included. We extracted 356 questions (refined to 271 by deduplication and relevance review), mapped items to Coalition for Health AI constructs, and organized them with iterative input from clinicians, patients, developers, epidemiologists, and policymakers.

**Results:**

We developed the Health Care AI Chatbot Evaluation Framework (HAICEF), a hierarchical framework with 3 priority domains (safety, privacy, and fairness; trustworthiness and usefulness; and design and operational effectiveness) and 18 second-level and 60 third-level constructs covering 271 questions. Emphasis includes data provenance and harm control; Health Insurance Portability and Accountability Act/General Data Protection Regulation–aligned privacy and security; bias management; and reliability, transparency, and workflow integration. Question distribution across domains is as follows: design and operational effectiveness, 40%; trustworthiness and usefulness, 39%; and safety, privacy and fairness, 21%. The framework accommodates both patient-facing and back-office use cases.

**Conclusions:**

HAICEF provides an adaptable scaffold for standardized evaluation and responsible implementation of health care chatbots. Planned next steps include prospective validation across settings and a Delphi consensus to extend accountability and accessibility assurances.

## Introduction

The rapid rise of chatbots, also known as conversational agents, has garnered substantial interest in the health care market. Valued at $787.1 million in 2022, the global health care chatbot market is expected to grow at an annual rate of 23.9% from 2023 to 2030 [1]. This expansion is driven by the increasing demand for virtual health assistance, growing collaborations between health care providers and industry players, and the acceleration prompted by the COVID-19 pandemic. For example, over 1000 health care organizations worldwide developed COVID-19-specific chatbots using Microsoft’s Health care Bot service to manage patient inquiries and reduce the burden on medical staff [2]. Entering the age of generative artificial intelligence (AI), health care chatbots have received even more attention since they enable human-level fluent conversations, have reached physician-level performance on board residency examinations [3] and comparable performance on other medical examinations and questions [4-6], and offer easy ways to train and adapt.

However, despite their popularity and potential, evaluating health care chatbots poses many challenges [7-9]. A lack of standardized evaluation approaches has led to diverse and inconsistent methods, making comparing chatbot performance difficult. Rapid technological advancements, particularly in generative AI, outpace existing regulatory frameworks [10], complicating the establishment of evaluation standards. These new chatbots using generative AI are not constrained by decision trees and are often built on top of larger models, meaning both the output and foundation are not stable. With such a moving target for evaluation, there is no widely accepted guideline or framework for evaluating health care chatbots. Developers lack a guide for assessment [11], and users often rely on company advertisements or marketing claims.

Several evaluation frameworks [12-22] have emerged in response to these challenges over the last few years, particularly following the popularity of generative AI. These frameworks vary—some review existing works and regroup metrics into a new structure, others adapt non–health care evaluation frameworks for this field, and some focus on narrow subdirections such as specific specialties or chatbot types. Given the need for a general guiding evaluation framework, a novel approach is necessary. Inspired by a framework for evaluating health apps [23], which has now been adopted by the American Psychiatric Association, we crafted a general evaluation framework integrating a literature review and broad stakeholder analyses. This approach involves the perspectives of developers, clinicians, patients, and policymakers to create a comprehensive evaluation structure.

## Methods

As health care chatbots face a variety of users, there is no single way to evaluate a chatbot. Factors such as safety and privacy, user preferences, technology literacy, accessibility, and treatment goals are crucial in determining the most suitable evaluation method. In addressing these issues, organizations like the Coalition for Health AI (CHAI) have been working on designing guidelines for trustworthy AI. In April 2023, a group of experts representing diverse stakeholders crafted a blueprint for trustworthy AI implementation guidance [24]. This blueprint includes 7 aspects of trustworthy AI in health care: usefulness, safety, accountability and transparency, explainability and interpretability, fairness, security and resilience, and enhanced privacy. But this framework serves more as a theoretical foundation rather than an empirical evaluation framework, and its similarity or overlap with other frameworks remains unclear.

Building on the construct definitions in this blueprint and existing evaluation frameworks, we (1) identified a total of 11 evaluation frameworks; (2) extracted all individual questions from these frameworks; (3) removed redundant and nonrelevant questions; (4) mapped the remaining questions to CHAI constructs, their subcategories, and constructs not covered by CHAI’s blueprint; (5) improved the evaluation framework structure with stakeholders, including health care providers, patients, technology developers, epidemiologists, and policymakers; and (6) further merged and rephrased questions based on assigned constructs.

**Figure 1:**
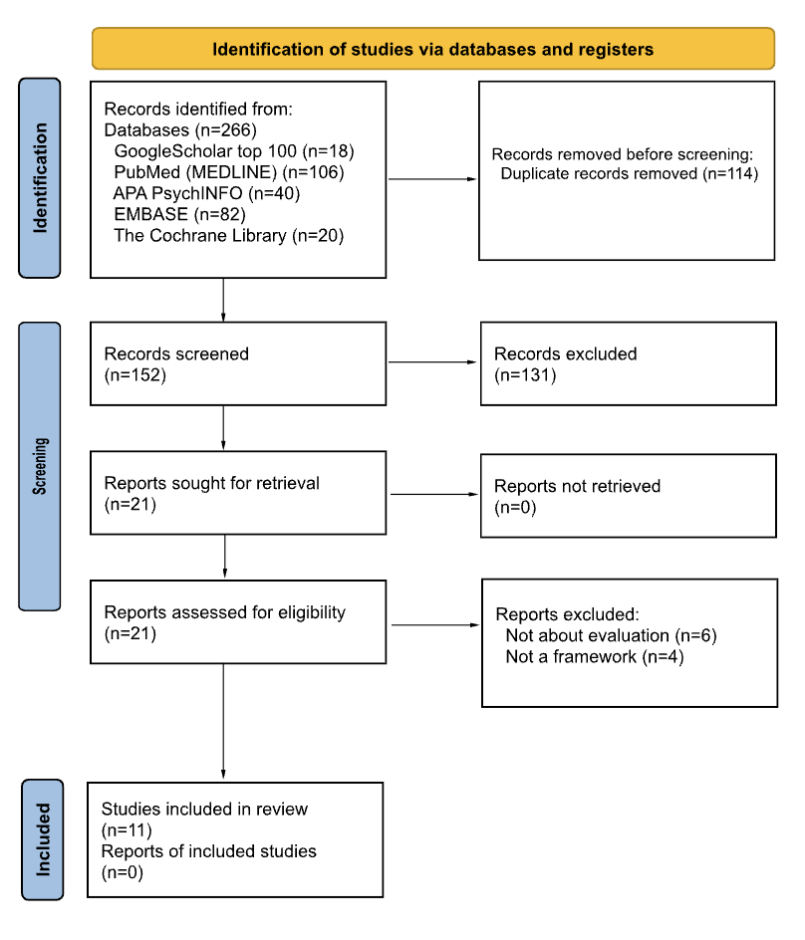
PRISMA (Preferred Reporting Items for Systematic reviews and Meta-Analyses) flow diagram of study selection for the evaluation of frameworks of health care conversational agents.

To identify and evaluate existing frameworks for health care conversational agents, we followed the PRISMA (Preferred Reporting Items for Systematic reviews and Meta-Analyses) guidelines to conduct a systematic review (Figure 1). The literature search was performed across multiple databases to ensure comprehensive coverage of relevant studies. The databases and corresponding search terms are shown in Table 1.

The initial search results were screened based on titles and abstracts. Two authors (YH and WX) independently reviewed the titles and abstracts for full-text retrieval, with any discrepancies resolved by discussion with a third reviewer (JT). Full-text articles were then retrieved for further assessment against the inclusion criteria. From the initial 266 records, 152 were screened, and 21 reports were sought for retrieval. After detailed assessment, 11 studies were included in the review, providing a comprehensive evaluation of frameworks for health care conversational agents.

We began by summarizing each framework’s intended use to assess specific concepts within a particular domain. The sections detailing the evaluation framework’s questions were then extracted and listed. If the study did not explicitly present evaluation criteria in the form of questions, these criteria were rephrased as questions for clarity. The following steps were taken:

1. Describe the use intention: the purpose and intended application of the framework were articulated, highlighting its relevance and scope.
2. Identify the concepts evaluated: the key concepts and dimensions the framework evaluates were identified and outlined.
3. List the evaluation questions: a thorough list of the questions evaluated by the framework was provided. In cases where the study did not present evaluation criteria as questions, these criteria were rephrased into question format for consistency and clarity.

**Table 1:** Search strategy for identifying relevant frameworks.

Database	Query
PubMed (MEDLINE)	(“health”[Title/Abstract] OR “medical”[Title/Abstract] OR “medicine”[Title/Abstract] OR “clinical”[Title/Abstract]) AND (“conversational agent”[Title/Abstract] OR “conversational AI”[Title/Abstract] OR “chatbot”[Title/Abstract]) AND (“framework”[Title/Abstract] OR “evaluation method”[Title/Abstract] OR “assessment method”[Title/Abstract])
EMBASE	(‘health’:ti,ab OR ‘medical’:ti,ab OR ‘medicine’:ti,ab OR ‘clinical’:ti,ab) AND (‘conversational agent’:ti,ab OR ‘conversational ai’:ti,ab OR ‘chatbot’:ti,ab OR ‘virtual agent’:ti,ab OR ‘virtual assistant’:ti,ab) AND (‘framework’:ti,ab OR ‘evaluation method’:ti,ab OR ‘assessment method’:ti,ab)
APA PsychINFO	(“health” OR “medical” OR “medicine” OR “clinical”) AND (“conversational agent” OR “conversational AI” OR “chatbot” OR “virtual agent” OR “virtual assistant” OR “digital assistant”) AND (“framework” OR “evaluation method” OR “assessment method”)
The Cochrane Library	(“health” OR “medical” OR “medicine” OR “clinical”) AND (“conversational agent” OR “conversational AI” OR “chatbot” OR “virtual agent” OR “virtual assistant” OR “digital assistant”) AND (“framework” OR “evaluation method” OR “assessment method”)
Google Scholar^a^	(“health” OR “medical” OR “clinical”) AND (“conversational agent” OR “conversational AI” OR “chatbot” OR “virtual agent” OR “virtual assistant” OR “digital assistant”) AND (“framework” OR “evaluation method” OR “assessment method”)

^a^Because Google Scholar does not support advanced search queries, we performed all combinations of searches separately to ensure comprehensive coverage.

To improve the evaluation framework structure, we adopted a similar approach to the 2019 study on creating a taxonomy for smartphone app evaluation [23]. This process involved a form of qualitative factor analysis, where all 4 authors, including 1 clinician, independently reviewed and discussed how the evaluation questions aligned with specific factors or categories. Consensus was reached through iterative discussions to ensure accuracy and relevance, allowing us to systematically organize the questions into meaningful constructs.

## Results

The reviewed frameworks are listed in Table 2 with details of their intended use, the concepts evaluated, and the terms used for conversational agents.

Initially, 275 questions were extracted from these frameworks (Multimedia Appendix 1). Questions that contained multiple subquestions were broken down for clarity. Broad questions that were too general to be useful were removed (eg, “Can strategies or solutions be developed to address problems of conversational agents?”). This iterative process led to a refined list of 271 questions, mapped across constructs outlined in the Coalition for Health AI blueprint and beyond (Multimedia Appendix 2).

**Figure 2:**
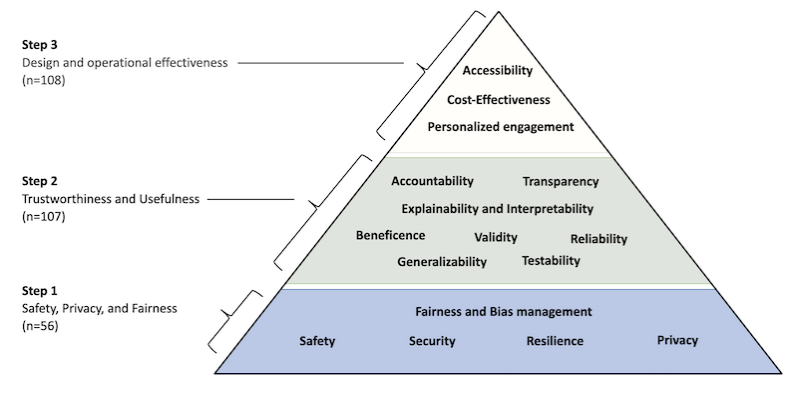
Pyramid visualization for the Health Care AI Chatbot Evaluation Framework (HAICEF; simplified, first 2 levels only). Priority-level constructs are displayed on the left, with second-level constructs within the pyramid.

The final structure of the Health Care AI Chatbot Evaluation Framework (HAICEF) represents 3 priority-level constructs, 18 second-level constructs, and 60 third-level constructs (the first 2 levels are shown in Figure 2 and the full framework is shown in Table 3). The 271 questions covered 56 third-level constructs. Among these questions, Design and Operational Effectiveness accounted for 108 (40%) questions. Trustworthiness and Usefulness accounted for a similar weight of 107 questions each (39%). The most fundamental level of Safety, Privacy, and Fairness included 56 questions (21%).

**Table 2:** Summary of reviewed framework studies for health care chatbots.

Title	Year	Term used for conversational agent	Intention	Reference
How to evaluate health applications with conversational user interface?	2020	Conversational user interface	Support evaluation of health systems using conversational user interfaces, define quality dimensions, and guide developers and researchers.	[12]
Conversational agents in health care: expert interviews to inform the definition, classification, and conceptual framework	2023	Conversational agent	Define and classify health care conversational agents, validate the a conceptual framework, and update another framework focusing on ethics, user involvement, and data privacy.	[16]
Developing a technical-oriented taxonomy to define archetypes of conversational agents in health care: literature review and cluster analysis	2023	Conversational agent	Develop a taxonomy of technical characteristics, identify archetypes, and harmonize evaluation metrics.	[14]
Evaluation framework for conversational agents with artificial intelligence in health interventions: a systematic scoping review	2023	Conversational agent	Propose a 4-stage evaluation framework (feasibility and usability, efficacy, effectiveness, and implementation) based on World Health Organization recommendations.	[21]
Evaluation of the current state of chatbots for digital health: scoping review	2023	Chatbot	Assess the current state of health-related chatbots, identify research gaps, guide future research, and enhance chatbot design.	[18]
Framework for guiding the development of high-quality conversational agents in healthcare	2023	Conversational agent	Provide a framework for the development and evaluation of health conversational agents, ensure patient safety, and ensure efficacy of conversational agent–delivered interventions.	[13]
Information quality of conversational agents in healthcare	2023	Conversational agent	Investigate definitions, influencing factors, and impacts of information quality in health conversational agents.	[15]
To chat or bot to chat: ethical issues with using chatbots in mental health	2023	Chatbot	Examine ethical issues in using chatbots in mental health, provide recommendations for ethical design and deployment.	[17]
Ethical Incorporation of Artificial Intelligence into Neurosurgery: A Generative Pretrained Transformer Chatbot-Based, Human-Modified Approach	2024	Chatbot, generative pretrained transformer	Delineate ethical considerations for AI^a^ in neurosurgery and present an ethical framework for AI integration.	[20]
Achieving health equity through conversational AI: a roadmap for design and implementation of inclusive chatbots in healthcare	2024	Conversational AI, chatbot	Develop a roadmap for inclusive conversational AI in health care and promote health equity.	[22]
Foundation metrics for evaluating effectiveness of healthcare conversations powered by generative AI	2024	Conversational AI, large language models	Establish a framework for evaluating the effectiveness of health care conversations using generative AI and address limitations of existing metrics.	[19]

^a^AI: artificial intelligence.

Table 3 presents the full structure of HAICEF, with definitions provided for each concept to enhance clarity. This review-based framework addresses multiple dimensions to ensure the effective, safe, and ethical operation of health care chatbots. For instance, safety considerations include measures such as data provenance, which ensures that the chatbot relies on credible data sources including hospital electronic health records and peer-reviewed studies, and harm control mechanisms to minimize risks, such as providing inaccurate advice that could lead to adverse outcomes. Privacy and security are critical pillars, with requirements for compliance with standards like the Health Insurance Portability and Accountability Act and the General Data Protection Regulation, robust data encryption practices, and clear privacy policies outlining how user data are handled. Fairness and bias management address systemic, computational, and population biases, ensuring that the chatbot provides equitable responses across diverse demographics. The emphasis on accountability and transparency mandates that chatbots clearly communicate their limitations, data usage, and performance metrics while adhering to ethical standards that prioritize patient safety and respect for autonomy. For example, questions such as “Is the chatbot compliant with data privacy regulations?” and “Does the chatbot avoid causing harm through incorrect information?” illustrate the necessity of rigorous evaluation to maintain trustworthiness and ethical integrity in health care applications (Multimedia Appendix 2).

**Table 3:** Framework for health care chatbot evaluation.

Constructs (levels 1, 2, and 3)				Description
**Safety, privacy, and fairness**				
	**Safety**		Prevent worse outcomes for the patient, provider, or health system from occurring as a result of the use of an ML^a^ algorithm.	
		Outcome proxies appropriateness	Use alternative measures or indicators that accurately reflect the desired health outcomes in the absence of direct measurements.	
		Data provenance	Track and document the origin and history of data, including where it came from and how it has been handled. Data providers: assign roles and responsibilities to entities like hospital EHRsb and patient-generated health data for maintaining safe AIc. Data sources: include various origins of data, such as social media and clinical settings.	
		Harm control	Reduce and manage potential risks and negative impacts associated with using a chatbot.	
		Automation bias reduction	The tendency to accept automated suggestions without critical evaluation or questioning.	
		Critical help	Provide necessary assistance and address negative and help-seeking information.	
		Ethics	Principles and standards that govern the conduct of individuals and organizations, ensuring fairness, privacy, and respect in using ML algorithms in health care.	
	**Security**		Maintain confidentiality, integrity, and availability through protection mechanisms that prevent unauthorized access and use	
		Protection method	Implement techniques and tools to safeguard data from unauthorized access and threats.	
		Security standard	Follow established guidelines and practices designed to protect data and systems from security breaches.	
		Third-party reliability	Ensure the trustworthiness of external partners or services in maintaining data security and integrity.	
	Resilience		Withstand unexpected adverse events or changes in their environment or use.	
	**Privacy**		Protect privacy according to standards like HIPAA^d^ and GDPR^e^, ensuring user autonomy and dignity.	
		Data exchange	Maintain privacy standards for accessing and sharing data with third-party tools, cloud platforms, and other external systems.	
		Data collection and storage	Maintain privacy standards for gathering and securely storing data for future use.	
		Data usage	Maintain privacy standards for using collected data for analysis, decision-making, and improving chatbot algorithms.	
		Privacy policy	Outline how an organization collects, uses, protects, and shares personal data.	
		Data protection	Implement methods to ensure privacy and prevent unauthorized access and breaches.	
	**Fairness and bias management**		Ensure the chatbots operate with minimized and acknowledged biases to ensure fair outcomes.	
		Systemic bias	Address biases originating from societal norms and institutional practices.	
		Computational and statistical bias	Manage biases arising from the way data is processed and algorithms are designed.	
		Human-cognitive biases	Recognize biases stemming from individual or group perceptions and attitudes.	
		Population bias	Address the issue where certain populations are underrepresented in data, leading to less accurate model performance for those groups.	
**Trustworthiness and usefulness**				
	Accountability		Ensure those involved in the chatbot’s lifecycle uphold standards of auditability and harm minimization.	
	**Transparency**		Communicate clearly regarding the chatbot’s characteristics and performance throughout its lifecycle.	
		Usage specification	Define how the chatbot should be used.	
		Model characteristics	Describe the specific features and behaviors of the chatbot**.**	
		Model availability	Ensure the chatbot is accessible as needed.	
		Model limitations	Identify and communicate the boundaries and constraints of the chatbot.	
		Data usage	Explain how data is used within the chatbot.	
	**Explainability and interpretability**		Described below.	
		Model explainability	Detail the internal mechanisms and decision-making processes of the chatbot.	
		Model interpretability	Make the outputs of chatbots clear and meaningful to end-users.	
	**Beneficence**		Ensure the chatbot positively impacts its intended outcomes, emphasizing measurable benefits over potential risks.	
		Health outcomes	Focus on improving health results.	
		Clinical evidence	Use rigorous methods like A/B tests or randomized controlled trials to validate effectiveness.	
		Use behavior	Influence and improve user actions.	
		Intervention	Apply targeted measures to achieve desired outcomes.	
		Health care system	Integrate effectively within the broader health care environment	
	**Validity**		Ensure the chatbot performs as expected in real-world conditions.	
		Data relevance and credibility	Use high-quality, pertinent training data.	
		Language understanding	Ensure the chatbot’s linguistic capabilities are robust.	
		Information retrieval accuracy	Accurately retrieve relevant information.	
		Outcome accuracy	Deliver precise and correct results.	
		Task completion	Effectively complete required functions.	
	**Reliability**		Ensure that the chatbot consistently performs as intended under various conditions and maintains dependable operation over time.	
		Failure prevention	Prevent system failures to maintain functionality.	
		Robustness	Handle unexpected inputs and diverse data without errors.	
		Workflow integration	Fit seamlessly into existing processes.	
		Reproducibility	Ensure consistent outcomes across different settings.	
		Monitoring	Continually check chatbots to ensure proper operation.	
		Up-to-dateness	Keep the system current with the latest information.	
	**Generalizability**		Apply learned patterns to new, unseen data.	
		Contextual adaptability	Function effectively in different environments or clinical contexts. Age group adaptability: cater to different age groups. Scenario adaptability: adapt to various situations.	
		Novel data performance	Perform well with new, unseen data.	
	**Testability**		Verify and meet standards for robustness, safety, bias mitigation, fairness, and equity.	
		Verifiability	Ensure different attributes can be tested. Quantifiability: measure attributes precisely.	
		Regular auditing	Measure attributes regularly.	
**Design and operational effectiveness**				
	**Accessibility**		Ensure the chatbot is usable by the intended users regardless of their abilities, devices, or technical skills, promoting inclusivity and ease of use.	
		Versatile access	Provide multiple interaction methods to accommodate user preferences and needs. Multilanguage: enable interaction in multiple languages to cater to a diverse user base. Different input and output modes: accommodate various input and output methods, such as text, voice, and visual. Multiplatform: ensure functionality across different platforms, such as web, mobile, and desktop applications. Multidevice: provide compatibility with various devices, including smartphones, tablets, laptops, and desktop computers.	
		User literacy	Ensure the system is usable by individuals with varying levels of technical knowledge and literacy.	
		User experience	Create a pleasant and effective interaction for users. Likability: design the system to be appealing and enjoyable to use. Understood by the conversational agent: ensure clear communication between the user and the chatbot. User engagement: maintain user interest and active participation. Respectfulness: interact with users in a polite and respectful manner. Response appropriateness: provide suitable and contextually relevant responses. Credibility: ensure the chatbot’s reliability and trustworthiness.	
		User interface design	Create an intuitive and easy-to-use interface for users.	
		Simplicity and ease of use	Make the system straightforward and user-friendly, minimizing complexity and effort required from users.	
	**Personalized engagement**		Tailor responses based on patient data and preferences.	
		Personalization	Customized responses based on patient data and preferences.	
		Anthropomorphism and relationship	Build a human-like relationship with users. Relationship building: develop a rapport with users. Empathy: show understanding and compassion. Humor: use appropriate humor to engage users. Identity: establish a clear and consistent chatbot persona.	
		User adherence	Track and analyze how well users follow recommendations and adjust the chatbot’s strategies based on this data to improve compliance and outcomes.	
		Feedback incorporation	Use user feedback to improve the system.	
		Progress awareness	Monitor and respond to the conversation’s context and progress. Memory: support multiturn or multisession conversations. Strategy adjustment: adapt the conversation strategy as needed.	
	**Cost-effectiveness**		Assess whether the chatbot delivers beneficial outcomes at a reasonable cost, providing a better or more economical solution compared to existing methods.	
		Comparative effectiveness	Demonstrate that the chatbot is a better solution than previous methods.	
		Economical viability	Ensure the system is cost-effective.	
		Environmental viability	Minimize environmental impact.	
		Task efficiency	Perform tasks quickly and effectively. Appropriate response time: provide timely responses. Response conciseness: give clear and succinct information. Response relevance: ensure responses are pertinent to the query. Response practicality: offer practical and actionable information.	
		Workflow considerations	Integrate smoothly into existing systems.	

^a^ML: machine learning.

^b^EHR: electronic health record.

^c^AI: artificial intelligence.

^d^HIPAA: Health Insurance Portability and Accountability Act.

^e^GDPR: General Data Protection Regulation.

**Figure 3:**
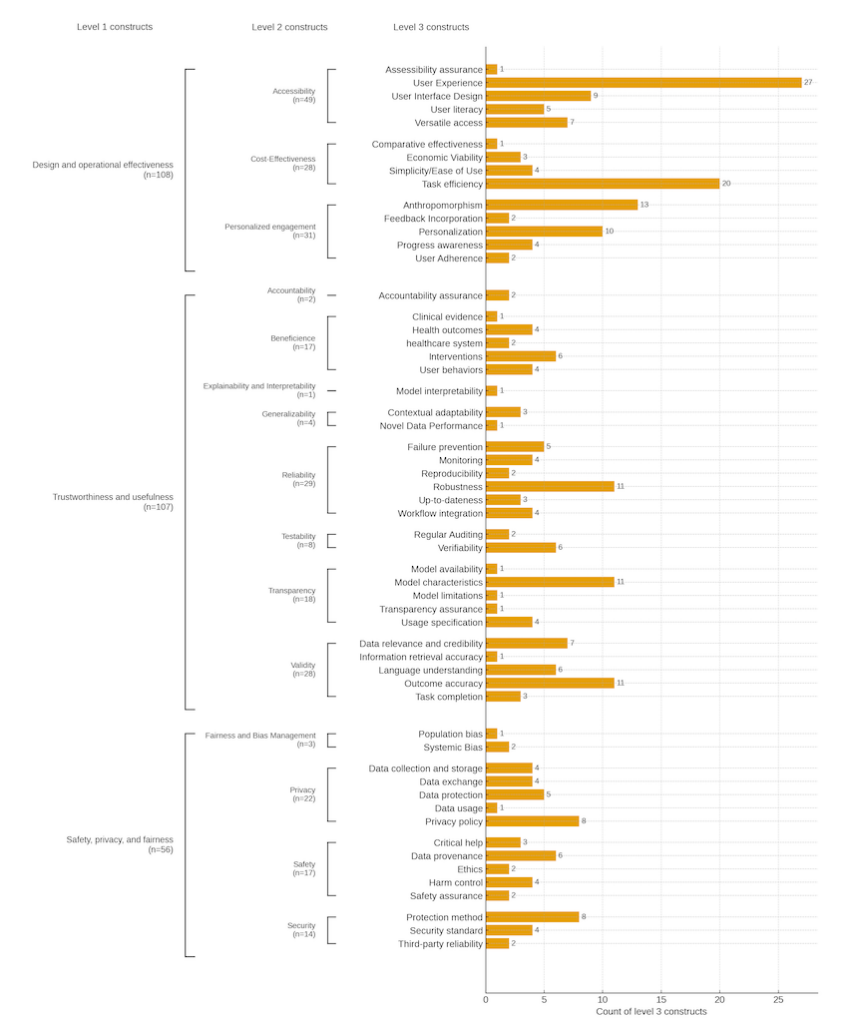
Distribution of constructs in the Health Care AI Chatbot Evaluation Framework (HAICEF) by level. This figure visualizes the distribution of level 3 constructs within HAICEF across its 3 hierarchical levels. The figure is organized by level 1 constructs (design and operational effectiveness, trustworthiness and usefulness, and safety, privacy, and fairness), with subdivisions showing their level 2 and level 3 components. The lengths of the bars represents the density of constructs within each dimension.

Figure 3 shows the distribution of constructs in the HAICEF framework, which shows a strong emphasis of current framework studies on “design and operational effectiveness,” accounting for 108 level 3 constructs, making it the most densely populated level 1 category. Within this, “accessibility” and “personalized engagement” dominate, with 27 and 20 level 3 constructs, respectively, reflecting the importance of inclusivity and tailored user interactions in chatbot evaluation. Conversely, “trustworthiness and usefulness” exhibits a balanced distribution across its level 2 constructs, such as “reliability” (11 constructs) and “transparency” (7 constructs), emphasizing the need for dependable and comprehensible chatbot systems. The “safety, privacy, and fairness” category, though less populous with 56 level 3 constructs, prioritizes critical areas like “privacy” (11 constructs) and “safety” (9 constructs), underscoring foundational principles necessary for secure and equitable operation.

In Table 4, we compare all reviewed frameworks across key evaluation dimensions, including safety, privacy, fairness, trustworthiness, and operational effectiveness. Each prior framework contributes valuable insights but remains partial, often focusing on isolated aspects rather than providing a comprehensive evaluation structure. HAICEF integrates these fragmented approaches, deduplicating and synthesizing 356 questions into a unified, adaptable framework that addresses the full spectrum of chatbot evaluation needs. By incorporating elements from all existing frameworks, HAICEF establishes the first standardized guidance for a holistic and comprehensive assessment across diverse health care applications.

**Table 4:** Comparison of reviewed health care chatbot evaluation frameworks and the Health Care AI Chatbot Evaluation Framework (HAICEF).

Construct		Framework references											
		[12]	[16]	[14]	[21]	[18]	[13]	[15]	[17]	[20]	[22]	[19]	HAICEF
**Safety, privacy, and fairness**													
	Safety	✔	✔		✔	✔			✔	✔	✔	✔	✔
	Security	✔	✔	✔		✔	✔				✔	✔	✔
	Resilience											✔	✔
	Fairness and bias management		✔						✔	✔	✔	✔	✔
**Trustworthiness and usefulness**													
	Privacy	✔	✔	✔		✔	✔		✔	✔	✔	✔	✔
	Accountability								✔	✔	✔		✔
	Transparency								✔	✔	✔		✔
	Explainability and interpretability								✔	✔		✔	✔
	Beneficence		✔						✔	✔			✔
	Validity	✔	✔		✔	✔	✔	✔			✔	✔	✔
	Reliability	✔				✔		✔				✔	✔
	Generalizability										✔	✔	✔
**Design and operational effectiveness**													
	Testability				✔		✔				✔	✔	✔
	Accessibility		✔				✔	✔			✔		✔
	Cost effectiveness		✔	✔							✔		✔
	Personalized engagement	✔	✔	✔	✔	✔						✔	✔

## Discussion

### Principal Findings

Chatbots are increasingly widely used in health care, but no comprehensive framework for evaluating their performance has been available. We surveyed the existing frameworks and developed a new framework using PRISMA guidelines, which we hope will facilitate future comparisons. The new proposed framework, HAICEF, is designed to meet the myriad users, use cases, and advances around health AI chatbots by providing a flexible scaffolding to support informed decision making.

HAICEF’s foundation in safety, privacy, and fairness is well aligned with recent research raising concerns about these aspects of chatbots. A 2024 review of AI apps concluded these apps may cause harm associated with bias [25], and the 2023 real-world case of an AI chatbot for eating disorders giving dangerous information to users [26] highlights the importance of step 1 in our framework (see Figure 2). Not all AI chatbots are patient-facing, and the framework is relevant to scaffolding conversations about clinical documentation chatbots, differential diagnosis chatbots, and even scheduling chatbots given the core aspects of the framework are relevant. For example, while efforts are underway to identify and address bias in conversational agents [27], checking for and identifying bias in any chatbot is a productive first step in considering any conversational agent and is a foundational step for avoiding harm.

Likewise, HAICEF’s second step, trustworthiness and usefulness, is grounded in recent research. From concerning trends of conversational agents drawing schizophrenia in a stigmatizing manner [28] to some chatbots providing details on self-harm and how to die by suicide [29], it is critical to assess the trustworthiness and usefulness of conversational agents. Given most conversational agents today are trained on social media, not health data [30], there is justified concern about the utility of the information provided. Additionally, subtle errors can be mixed with correct responses that are difficult for even experts to detect [31]. While there are many approaches to determine trustworthiness and usefulness, and our framework does not dictate which should be used, the structure ensures a focus on this critical issue.

HAICEF also celebrates the success of conversational agents with step 3 considering factors like their often-high degree of accessibility and efforts to personalize content. In placing step 3 after the prior 2, our framework reminds the user to first consider the potential risks and appropriateness of the conversational agent. The majority of frameworks we assessed (Table 4) focused on the questions included here in step 3. Our approach provides a complimentary means to consider these same questions but in the broader context of steps 1 and 2.

The framework offers several advantages by synthesizing insights from previous efforts into a new, synergistic model applicable across diverse health conditions and stakeholder groups. Unlike traditional methods that report isolated metrics, HAICEF re-evaluates existing frameworks to distill and integrate them into a comprehensive general guiding framework. It is not designed to challenge or replace any framework and is flexible enough to incorporate new ones that will likely be developed.

A distinctive feature of the framework is its multilevel tree structure, mapping questions into granular constructs without assigning scores to individual questions. This approach facilitates future development of more detailed, domain-specific evaluation methods, using our framework as a reference or guide. Additionally, we aimed to maintain a consistent level of granularity across all levels of the framework, ensuring that each aspect of evaluation is addressed with equal thoroughness.

The pyramid structure, similar to Maslow’s Hierarchy of Needs [32], serves as a visual reminder that evaluation may begin at the base, and progression is likely unnecessary if any level fails to meet the required standards. Still, the user may opt to approach the constructs and questions in any manner that suits their needs. The process of going through these questions will likely facilitate productive dialogue and reveal tensions that must be addressed by the user to make the optimal selection. Thus, this structure does not itself perform an evaluation but rather serves as a scaffold for evaluation. The same chatbot will be evaluated differently depending on the user and their intent for use, reflecting the flexible nature of this framing. The detailed questions, summarized in Multimedia Appendix 1, are designed to encourage and facilitate dialogue among stakeholders, with responses contextualized within each stakeholder’s unique situation. For instance, some chatbots may collect user conversation histories for training purposes by default. Some patients may find this unacceptable, while others may be comfortable with it. Similarly, developers focused on improving chatbot validity and reliability should not be compelled to conduct user feedback field studies if their research scope explicitly excludes user experience.

The rise of generative AI, such as ChatGPT [33], has expanded interest in health care chatbots, placing a pressing need for robust evaluation guidance. Yet the emergence of so many frameworks may create more uncertainty. By assessing the details of numerous frameworks, we were able to simplify and unify different approaches to help inform decision-making. The current framework is designed to be flexible and serve various decision makers—from a designer seeking to create a new chatbot to a patient selecting one from the marketplace. Depending on the user and use case, a different weighting to each construct will be necessary, in the same manner that ethical principles offer a scaffold to guide diverse decision making. Our analysis (see Figure 3) suggests that while most frameworks emphasize factors like user experience and task efficiency, stakeholder feedback indicates that focusing on safety and usefulness (see Figure 2) may better match user needs and concerns. Furthermore, we hope these findings will help guide policymakers to design effective evaluation regulations for health care chatbots, safeguarding the quality of information and providing a clear roadmap for businesses worldwide to further develop tools that improve care.

Finally, certain constructs, such as accessibility assurance and accountability assurance, remain placeholders due to limited coverage in the literature. These elements underscore the need for further investigation to develop more specific evaluation questions and classifications. As research on these areas grows, we anticipate integrating new findings into an expanded framework that remains both comprehensive and adaptable.

This approach has several limitations. The framework should be validated prospectively in different contexts to ensure that it is comprehensive and captures important dimensions. There may be additional dimensions that need to be added as the underlying technology quickly evolves, uncovering new issues. Another limitation pertains to the selection and appraisal of source articles. We did not perform a formal quality assessment of each included framework beyond ensuring they met the inclusion and exclusion criteria (eg, scope and relevance to health care chatbots). All frameworks that aligned with these criteria were deemed acceptable. We justified this approach in order to cast a broad net and capture the wide diversity of existing evaluation tools. However, future iterations of this review and framework could benefit from a standardized critical appraisal of each source to more rigorously assess the quality of the evidence guiding HAICEF.

Given the absence of a universal standard for evaluating health care chatbots, many parallel review tools have emerged, often failing to capture the full range of important considerations. Our framework addresses this gap, offering a comprehensive, adaptable tool for the evaluation of health care chatbots, which we hope will lead to responsible integration of chatbots into health care settings. Furthermore, we hope that this review will help guide policymakers to design effective evaluation regulations for health care chatbots, both to safeguard the quality of information and provide a clear roadmap for businesses worldwide to further develop tools that improve the quality, efficiency, and effectiveness of care.

HAICEF presents a starting point that will evolve. Next steps include fully exploring the needs of different users of health AI chatbots and their most common intents and goals. Exploring chatbots beyond the classical medical domains (eg, nephrology and radiology) and understanding functions across the health care ecosystems (from scheduling to crisis support) will help ensure the framework is responsive to real-world needs. Further work to expand the granularity of individual questions and their focus on users (eg, developers versus clinicians) will help improve usability. Additionally, we plan to engage a broader range of stakeholders through a Delphi consensus process to ensure the framework’s inclusivity and adoption. These efforts aim to establish HAICEF as a rigorous and widely adopted standard, enabling consistent and comparable evaluations across the health care sector. Through these efforts, we hope to establish a more rigorous, inclusive, and widely adopted evaluation framework for health care chatbots and enable “apples to apples” comparisons between them.

### Conclusions

This is the first work to develop a structured and adaptable framework for evaluating health care AI chatbots, addressing the urgent need for standardized assessment criteria. By synthesizing insights from existing frameworks and diverse stakeholders, we developed a structured approach that prioritizes safety, privacy, trustworthiness, and usefulness. This framework is intended to guide the responsible evaluation and implementation of chatbots in health care, helping to ensure their safe and effective use. Future work will focus on validating and refining this framework in different contexts.

## Appendix

Multimedia Appendix 1 Extracted questions.

Multimedia Appendix 2 Mapped Framework Questions.

## Abbreviations

AI: artificial intelligence
CHAI: Coalition for Health AI
HAICEFL: Health Care AI Chatbot Evaluation Framework
PRISMA: Preferred Reporting Items for Systematic reviews and Meta-Analyses
